# Exploring sexual and romantic functioning as early risk factors of schizophrenia: a narrative review

**DOI:** 10.1093/sexmed/qfaf002

**Published:** 2025-02-06

**Authors:** Paula Dospělová, Petra Šustová, Ellen Zakreski, Renáta Androvičová

**Affiliations:** First Faculty of Medicine, Charles University, Kateřinská 1660/32,121 08, Prague, Czech Republic; Centre for Sexual Health and Interventions, National Institute of Mental Health, Topolová 748, 250 67, Klecany, Czech Republic; Centre for Sexual Health and Interventions, National Institute of Mental Health, Topolová 748, 250 67, Klecany, Czech Republic; Faculty of Arts, Charles University, nám. J. Palacha 1/2, 116 38, Prague, Czech Republic; Centre for Sexual Health and Interventions, National Institute of Mental Health, Topolová 748, 250 67, Klecany, Czech Republic; Faculty of Humanities, Charles University, Pátkova 2137/5, 182 00, Prague, Czech Republic; Centre for Sexual Health and Interventions, National Institute of Mental Health, Topolová 748, 250 67, Klecany, Czech Republic; Genetic Epidemiology, Department of Psychiatry, Amsterdam University Medical Centers, University of Amsterdam, Meibergdreef 5, 1105 AZ, Amsterdam, the Netherlands

**Keywords:** schizophrenia, psychosexual development, sexual behavior, gonadal steroid hormones, sexual trauma, sexual dysfunction

## Abstract

**Background:**

Patients with schizophrenia frequently encounter challenges related to sexuality and intimacy; however, the underlying causes of these difficulties remain unknown and unexplored.

**Aim:**

This narrative review aims to explore how the biological/hormonal and psychological/behavioral developmental trajectories in schizophrenia patients deviate from the normal course and to examine their connection to difficulties in sexual and romantic functioning.

**Methods:**

A comprehensive literature search was conducted using PubMed and Google Scholar, with key terms related to schizophrenia and sexual development without restriction on publication year. Articles discussing behavioral, sexual, or psychological/behavioral development before the onset of schizophrenia were included. Articles were divided into biological/hormonal and psychological/behavioral precursor categories. Additional searches were conducted to explore the broader sociocognitive context of schizophrenia, such as deficits in empathy, emotional processing, and theory of mind.

**Outcomes:**

The review highlights deviations in both biological/hormonal and psychological/behavioral development in schizophrenia that contribute to difficulties in romantic and sexual relationships.

**Results:**

This narrative review addresses the extent to which biological, psychological, and social factors in schizophrenia may be closely intertwined. Abnormalities in the hypothalamic–pituitary-gonadal and hypothalamic–pituitary–adrenal axes have been documented in individuals with schizophrenia, potentially impairing sociosexual competencies and leading to behavioral challenges in forming sexual relationships. Deficits in theory of mind, emotional processing, and empathy may further hinder the ability to form and sustain intimate relationships, amplifying the social difficulties associated with schizophrenia. Additionally, early life traumas, especially sexual abuse, can contribute to sexual difficulties and worsen the disorder.

**Clinical Translation:**

Understanding the deviations from the normal developmental course in schizophrenia patients may offer valuable insights for potential intervention strategies and remediation approaches and contribute to improvements in sexual/romantic functioning and overall sexual health in schizophrenia patients.

**Strengths and Limitations:**

This review provides an overview of the developmental precursors of schizophrenia-related sexual/romantic difficulties. Further research is needed to elucidate the specific mechanisms underlying these difficulties, particularly in determining the emotional and motivational salience of sexual stimuli and the capacity to engage in and maintain communication of sexual interest. The reader should bear in mind that narrative reviews lack systematic methods for selecting and evaluating studies, which can lead to author bias in choosing or interpreting sources.

**Conclusion:**

The narrative review identified deviations in the biological/hormonal and psychological/behavioral developmental trajectories of schizophrenia patients, linking these abnormalities to difficulties in sexual and romantic functioning, and highlighting the need for sexological remediation strategies to improve sociosexual competencies and overall sexual health.

## Introduction

Although affecting less than 0.5% of the population,^1^ schizophrenia stands as the fifteenth leading cause of disability worldwide,^2^ with its global disease burden increasing by 62% between 1990 and 2017.^3^ The treatment of schizophrenia is challenging; although various interventions exist, full remission is achieved in only a minority of patients (22%).^4^ Pharmacological interventions, while crucial, can only mitigate 13% of the disease burden, potentially due to the lack of personalized therapeutic approaches.^5^ Despite the overwhelming nature of psychotic episodes, the primary disease burden stems from impaired social functioning and persistent negative symptoms, which seldom remit with antipsychotic treatment alone.^6^ In the area of social functioning, issues related to sexual and reproductive satisfaction stand out as paramount concerns for patients,^7^ with significant implications for treatment adherence.^8^

The relationship between schizophrenia and sexuality is both distinct and underexplored, with several notable intersections. First, the incidence of early trauma, including sexual abuse, is notably higher among patients with schizophrenia compared to healthy individuals.^9^^,^^10^ Childhood sexual abuse increases the risk of transitioning to psychosis^11^^,^^12^ and affects the development of sexuality and intimacy needs.^13^ Second, the onset of schizophrenia often coincides with the beginning of the reproductive period, typically occurring in late adolescence and early adulthood, with a peak age of onset at 20.5 years.^14^ Additionally, drug-naïve patients may exhibit lower levels of sex hormones and neurosteroids.^15^^,^^16^ Impaired sexual functioning is observed even before the onset of psychosis and prior to the beginning of treatment.^17^ Last, individuals with schizophrenia have fewer offspring and lower marriage or cohabitation rates.^18-20^ Interestingly, sexuality often becomes a topic of delusional ideation, covering themes of sexual identity, jealousy, and concerns about genital functioning.^21^

Evidence suggests that both biological/hormonal and behavioral aspects of reproductive maturity in patients with schizophrenia may be affected even prior to antipsychotic medication and may be related to the disease etiology and outcome.^16^ This aligns with the conceptualization of schizophrenia as a neurodevelopmental disorder where neuropathological triggers are in place much earlier than symptoms required for clinical diagnosis.^22^

Numerous studies have emphasized and examined the frequently unmet needs in intimate and sexual relationships among patients with schizophrenia.^23-25^ While studies demonstrate that patients with schizophrenia express a desire for sexual and romantic connections,^26^^,^^27^ they face challenges in establishing and maintaining such relationships.^23^^,^^24^

The underlying causes of deficits in sexual and romantic functioning remain unknown and unexplored. Sexual and romantic interaction emerges as a promising marker in patients with schizophrenia, representing a unique class of risk factors that may offer insights into the progression of the disorder and its impact on interpersonal functioning.

In this narrative review, we aim to:

1. Identify the biological, psychological, and social precursors of the capacity to establish a romantic/sexual dyad;
2. Explore how biological/hormonal and psychological/behavioral trajectories in patients with schizophrenia deviate from the normal developmental course in the context of romantic/sexual functioning;
3. Discuss the potential underlying mechanisms of sexual/romantic difficulties in patients with schizophrenia.

## Methods

To explore this issue, we identified and included relevant literature through a multi-step process. First, we conducted a literature search using PubMed and Google Scholar to identify key biological, psychological, and social precursors of the capacity to establish romantic relationships. Based on the search results, we have identified the main areas we will focus on within the scope of our research topic. Next, we searched PubMed and Google Scholar using combinations of terms related to schizophrenia (as Medical Subject Headings, ie, MeSH, terms or text words) and sexual development (in Title/Abstract), without any restriction on publication years. We included articles that discussed behavioral, sexual, or psychological/behavioral development prior to the onset of schizophrenia and excluded those irrelevant to the focus of this paper. We then screened the references of the included articles using the same procedure. All relevant articles published in English were reviewed.

We categorized papers directly relevant to the focus of this review into respective sections (biological vs. psychological/behavioral precursors). Additional literature searches in PubMed and Google Scholar were performed using key terms such as “schizophrenia,” “first-episode,” “ultra-high risk,” “psychosis,” “prodromal, ““puberty,” “adolescent,” “sexuality,” “sexual behavior,” “romantic relationships,” “romantic functioning,” “sexual relationships,” “sexual dysfunctions,” “gonadal steroid hormones,” “sex hormones,” “testosterone,” “estrogen,” “hypothalamic–pituitary-gonadal axis,” “hypothalamic–pituitary–adrenal axis,” “sexual trauma,” “sexual abuse,” “social functioning,” “cognitive development,” “empathy,” “theory of mind,” and “emotion processing,” including their combinations. The reference lists of all retrieved articles were also examined for further relevant studies. A total of 131 publications were included in the review.

As a narrative review should allow for the broadest possible approach, studies of any design and methodological quality were included. However, we prioritized studies with larger sample sizes and incorporated the findings of systematic reviews and meta-analyses when available. We acknowledge that the narrative review approach carries a higher potential for bias compared to systematic reviews. However, we have strived to be inclusive and objective in our selection process. As a result, this narrative review provides qualitative rather than quantitative insights into the complex relationship between schizophrenia and sexual/romantic functioning.

## The ability to establish a sexual/romantic dyad as a key outcome of adolescence

Adolescence is a crucial period for the establishment of sexual and romantic relationships, which are impacted by the developmental disruptions seen in schizophrenia.^16^ According to Sisk & Foster,^28^ reproduction is a developmental priority and a milestone of emerging adulthood. It requires the fulfillment of two conditions: (1) optimal function of the hypothalamic–pituitary-gonadal (HPG) axis, which secures gamete maturation, and (2) behavioral means for bringing male and female gametes together.

Reproduction is regulated via hormones of the HPG axis. At puberty onset, high-frequency gonadotropin-releasing hormone (GnRH) release activates GnRH neurons, triggering gametogenesis and increased gonadal steroid hormone secretion.^29^ These hormones then remodel and activate neural circuits, leading to the development of sexual salience of sensory stimuli, sexual motivation, and expression of copulatory behaviors in specific social contexts.^28^

The increases in estrogen and testosterone during adolescence enhance goal-oriented behavior related to sexual and romantic relationships.^30^ Researchers have found that testosterone is associated with mating effort, or people’s motivation to find a sexual partner,^31^^,^^32^ estradiol is correlated with an increase in sexual desire, while progesterone is linked with declines in sexual motivation.^33-35^ Many individuals with decreased sexual desire also experience endocrine dysregulation, such as reduced plasma testosterone levels and hyperprolactinemia.^36^ Hormonal balance in the brain is crucial to the development of sociosexual competences, and biological changes during puberty also organize neural circuits for adult social and reproductive behaviors.^28^^,^^37^

Basic social detection and theory of mind develop in early childhood, whereas many sociocognitive processes such as mentalizing, metacognition, and emotional regulation mature and develop during adolescence.^38^^,^^39^ The health and quality of romantic relationships are also influenced by adolescent interpersonal skills like assertiveness and positive engagement,^40^ the skills of insight, empathy, and emotional regulation.^41^ Interpersonal experiences in early life play a significant role in shaping the development of fulfilling romantic relationships.^42^ The quality of caregiving provided by parents predicts an individual’s ability to establish and maintain romantic relationships as an adult.^38^^,^^43^ It has been documented that destructive interparental conflict^44^ and interpersonal trauma exposure^45^ are risky for emerging adult’s romantic experience. On the contrary, supportive peer networks positively affect the timing and stability of romantic relationships.^46^

## How do biological/hormonal trajectories in patients with schizophrenia deviate from the normal developmental course?

A wide range of atypical physiological patterns are associated with schizophrenia, including altered regulation of sex hormones, inflammation, metabolism, and many other processes. The neuroendocrine systems regulating sex and stress hormones are particularly significant due to their impact on brain development and function and their influence on other related physiological changes, such as inflammation and metabolism.^47-49^ These two key neuroendocrine systems are the HPG axis, which regulates sex steroids, including androgens (eg, testosterone, dehydroepiandrosterone – DHEA) and estrogens (estradiol being the most physiologically active), and the hypothalamic–pituitary–adrenal (HPA) axis, which regulates hormones that play a critical role in the stress response (eg, corticotropin releasing hormone – CRH, adrenocorticotropic hormone – ACTH, and cortisol).

Schizophrenia has been linked to abnormal function of the HPG axis. Estrogen deficiency in women and testosterone deficiency in men were observed in antipsychotic-free patients with schizophrenia.^50^ Lower levels of testosterone were found in adolescent males with prodromal symptoms compared to healthy males,^51-53^ as well as in patients with first-episode schizophrenia after 6 months of antipsychotic treatment.^53^

The later onset of schizophrenia in women is suggestive of the protective role of estrogen. According to Riecher-Rössler,^54^ Seeman,^55^ and Cechnicki et al.,^56^ the production of physiologically high estradiol in premenopausal women delays the onset and improves the course of schizophrenia in women up until menopause. Later rise of estrogen levels seems to be associated with an earlier onset of the disorder.^57^ Estrogen levels in patients with first-episode schizophrenia were found to be lower than normal limits,^50^ even prior to the introduction of antipsychotic medication.^58^ Women with first-episode psychosis have reported gonadal dysfunction before the onset of the disease, mid-cycle bleedings, loss of hair, hirsutism, and infertility more often than a control group of age-matched healthy women.^59^

In addition to atypical HPG-axis activity, alterations of the HPA axis are also present in schizophrenia and psychosis. Cortisol, the primary effector of the HPA axis, plays a critical role coordinating the physiological stress response but also regulates metabolism, inflammation, brain function, and development.^60^^,^^61^ Excessive activation of the HPA axis, due to chronic stress, genetics, or other factors, leads to disease and induces subsequent dysregulation of the HPA axis.^62^^,^^63^ Various signs of HPA axis dysregulation have been observed in schizophrenia and psychosis. For instance, elevated levels of unstimulated cortisol have been observed in first-episode psychosis^49^ and schizophrenia,^64^ with particularly high levels observed in untreated schizophrenic patients.^64^^,^^65^ Elevated cortisol levels increase dopaminergic signaling throughout the brain, including the mesolimbic system.^66^ Increased mesolimbic dopamine transmission in turn contributes to the formation of positive symptoms.^67^ The dopaminergic pathway plays a crucial role in motivation and sexual drive. Striatal dopaminergic dysregulation is also linked to negative and cognitive symptoms that interfere with the neural processing of reward-predicting cues, thereby reducing motivational drive.^17^

Elevated cortisol levels may also interfere with the growth and maintenance of neurons. For instance, elevated cortisol inhibits hippocampal neurogenesis.^68^ Using post-mortem tissue, Issa et al.^69^ found abnormally high levels of cortisol in the prefrontal cortex in schizophrenic patients, and those higher levels of cortisol were, in turn, significantly associated with lower levels of brain-derived neuroprotective factor (BDNF). While HPA dysregulation may precipitate the onset and morbidity of schizophrenia, untreated schizophrenia may lead to excessive levels of stress, leading to HPA dysregulation. This could partly explain why cortisol levels decrease following antipsychotic treatment.^65^ Further research is however needed to explore this.

## How do psychological/behavioral trajectories in patients with schizophrenia deviate from the normal developmental course?

Social adjustment prior to the disease onset seems to be markedly affected in schizophrenia, including social isolation and poor peer relationships during childhood and adolescence.^70^ Poor premorbid social functioning (such as contact with other children, forming friendships, and participating in social activities), as reported by teachers, has also been identified as a predictor of adult schizophrenia-spectrum disorders.^71^ Social functioning, therefore, appears to serve as an early risk factor of illness vulnerability but also functions as a chronic stressor, potentially exacerbating the course of the illness. Age appears to influence the strength of the association; for example, Tarbox and Pogue-Geile^72^ found that poor undifferentiated social functioning beginning around age 7-8 may moderately predict schizophrenia in the general population.

Different trajectories in premorbid social functioning have been observed: some patients exhibit poor social adjustment such as difficulties with sociability, peer relationships, academic performance, and adaptation to school already in childhood, while others begin to deviate from their healthy peers during adolescence.^73^ Social isolation itself is considered a vulnerability factor for schizophrenia^74^ and is also one of the early signs of the prodromal phase of psychosis.^75^ The prodromal phase is characterized by a range of mental state features, including nonspecific symptoms like anxiety and depressed mood, along with subthreshold or attenuated psychotic symptoms. Because the prodromal stage can only be identified retrospectively, subjects exhibiting prodromal symptomatology are categorized prospectively as ultra-high risk.^76^

Social isolation typically precedes the onset of psychosis by 2-4 years and serves as a predictive factor for a first psychotic episode. For instance, Cornblatt et al.^74^ found that adolescents who later developed psychosis had fewer than two friends, preferred small social groups, and did not have a romantic partner.

In patients with schizophrenia, behavioral and psychological precursors are often shaped by early life experiences, particularly social factors such as interpersonal trauma and sexual abuse. Research suggests that early traumas and attachment experiences significantly influence adult attachment styles, which, in turn, affect the quality of future relationships.^77-79^ For example, exposure to violence in childhood can contribute to the development of anxious and avoidant attachment styles,^77^^,^^80^ both of which are more prevalent in individuals with psychotic disorders.^81^ Insecure attachments often stem from early traumas and adverse childhood experiences.^77^

The literature consistently indicates that the prevalence of early trauma is significantly higher in schizophrenia patients compared to healthy individuals^9^^,^^82^ or other patient groups.^83^^,^^84^ This suggests that early trauma increases risk of developing schizophrenia,^9^^,^^85^^,^^86^ alongside forming insecure attachments.^77^

The most frequent type of early trauma in schizophrenia patients is emotional neglect and emotional abuse, usually followed by physical neglect, physical abuse, and sexual abuse,^9^^,^^84^^,^^87^ and the occurrence of one type might potentially alter the impact and consequences of other types.^83^ There is also a dose–response relationship between early trauma and schizophrenia, that is, the more severe the early trauma, the more severe the symptoms of schizophrenia.^82^

Although the sexual trauma is not the most prevalent type of early trauma, it differs from other types in a significant way: when physical and emotional early trauma occur infrequently, it does not necessarily increase the risk of schizophrenia, but only one experience of sexual abuse increases the odds and severity of schizophrenia.^88^ Sexual trauma therefore plays a different role in the development of schizophrenia than emotional or physical abuse in many ways. First, sexual trauma is correlated with the development of anxious attachment in schizophrenia patients.^77^ Second, sexual trauma is correlated with lifetime suicidal attempts in schizophrenia patients.^86^^,^^89^ Sexual trauma is, furthermore, correlated with more severe positive symptoms as well as with occurrence of sexual hallucinations.^86^^,^^90^ And last, sexual trauma increases the risk of developing psychosis itself.^11^

Bell, Foulds, Horwood, Mulder, and Boden^91^ suggest that the impact of child sexual abuse is linked to alterations in emotional, cognitive, and neurobiological processes, including heightened emotional reactivity, poor emotion regulation, and impaired cognitive control. These changes may involve impacts on biological systems (eg, dysregulated cortisol levels) and psychological processes (eg, source monitoring biases, dissociation, and cognitive schema/thinking styles).

Child sexual abuse is also a significant risk factor for developing sexual dysfunction and intimacy issues in adulthood.^92^^,^^93^ According to de Jager et al.,^13^ child sexual abuse may contribute to unmet sexuality and intimacy needs in individuals with psychosis spectrum disorders.

## Sexual and romantic difficulties as a specific early risk factor of schizophrenia

Sexual/romantic difficulties are sometimes a priori subsumed under a wider sociocognitive and neurocognitive disability, but several studies have found that sociosexual deficits are a specific class of risk factors. Dragt et al.^76^ followed 72 ultra-high-risk individuals for developing psychosis over a period of 36 months. Of the 18 items from a widely used Premorbid Adjustment Scale questionnaire (PAS),^94^ social-personal adjustment and social-sexual aspects were identified as significant predictors for transition into psychosis. Salokangas and Stengård^95^ found major difficulties associated with heterosexual development in patients with schizophrenia, with men showing greater difficulties than women (only 52% of men as compared to 75% of women had an established relationship with the opposite sex by the age of 23 years).

Several qualitative studies have found that stress linked to interactions with the opposite sex, navigation of courtship situations, establishment of a sexual partnership, and the related processes of individuation from family are all issues which patients often perceive as precipitating factors of disease onset.^96-98^ Not only do patients with schizophrenia exhibit sociosexual difficulties, but so do their first-degree relatives. Research has shown that children and siblings of patients with schizophrenia report impairments in romantic relationships and more frequent problems with members of the opposite gender during adolescence and young adulthood.^99^ Hans et al.^100^ investigated the social interactions (with both same-sex and opposite-sex peers) of children whose parents had schizophrenia, other mental disorders, or no mental disorders. They discovered that children of schizophrenic parents had poor engagement with opposite-sex peers, but not with same-sex peers. In contrast, children of parents with other mental disorders and those of healthy parents showed similar levels of engagement with opposite-sex peers.

The Finnish Adoptive Family Study of Schizophrenia^101^ revealed significant social functioning deficits among offspring at high risk for schizophrenia spectrum disorders during adolescence. Compared to high-risk females, high-risk males showed significant deficits in their romantic relationships. These results indicate that social functioning deficits during adolescence may be linked to genetic susceptibility to schizophrenia-spectrum disorders, and that some of these deficits might be specific to gender.

A specific class of sexual problems in schizophrenia is sexual dysfunction. While there is substantial evidence of sexual dysfunction as a consequence of antipsychotic treatments,^102-104^ there is also emerging evidence of sexual dysfunction as an early risk factor of schizophrenia prior to antipsychotic medication.^17^^,^^105^^,^^106^ According to a systematic review and meta-analysis of 72 studies on sexual dysfunction in medicated individuals with schizophrenia, the overall prevalence of sexual dysfunction is 56.4%, with rates of 55.7% in men and 60.0% in women.^107^ The most common sexual dysfunction is erectile dysfunction (44% of men), followed by loss of libido (41%), ejaculation dysfunction (39% of men), orgasm dysfunction (28%), and amenorrhea (25% of women). This study indicates that the prevalence of sexual dysfunction remains high among individuals with schizophrenia, with no significant improvement over time or better tolerance of second-generation antipsychotics.^107^

Markedly fewer studies have focused on sexual dysfunction among individuals at ultra-high risk of psychosis or unmedicated patients compared to those examining medicated patients. A systematic review of five studies showed that the prevalence of sexual dysfunction in psychotic unmedicated patients ranged from 16.8% to 70%, while ~50% of individuals at ultra-high risk of psychosis were affected.^17^ The impairment seems to impact all sexual domains, from desire to orgasm. Sexual desire dysfunction ranges from 14% to 20%, sexual arousal dysfunction varies between 4% and 60%, and orgasm dysfunction ranges from 1% to 56%. The same study found systematically higher rates of sexual dysfunction among psychotic and ultra-high-risk patients compared to the healthy population. The authors have also suggested that psychotic symptoms and sexual dysfunction may share common underlying causes at both psychosocial and neurobiological levels. A study by Marques et al. revealed that individuals at ultra-high risk of psychosis and unmedicated patients experiencing a first episode of psychosis exhibit worse sexual functioning compared to healthy individuals.^105^ According to the authors, sexual dysfunction appears to be a characteristic of vulnerability to, or development of, psychotic illness rather than an adverse effect of antipsychotic medication. Further investigation is warranted to address the causes of sexual dysfunction in unmedicated patients.

## Possible sources of sexual and romantic difficulties

**Figure 1:**
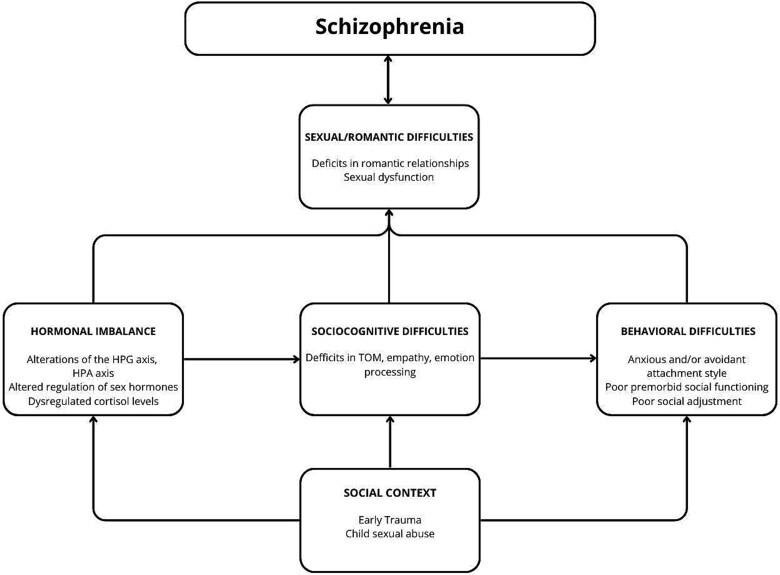
Developmental trajectories contributing to sexual and romantic difficulties in schizophrenia patients.

The sexual and romantic deficits outlined above can be attributed to multiple factors. We have developed a conceptual map (Figure 1) to illustrate some of the complex relationships between these factors and the sexual developmental difficulties experienced by patients with schizophrenia.

As discussed in this paper, the development of individuals with schizophrenia differs from the healthy population both biologically/hormonally and psychologically/behaviorally. Hormonal balance in the brain and periphery plays a crucial role in the development of sociosexual competencies, potentially contributing to behavioral difficulties in forming sexual relationships.^28^ The behavioral difficulties in establishing a sexual/romantic relationship might be linked to impairments in sociocognitive abilities (such as deficits in theory of mind, emotion processing, and empathy), which can manifest as impaired ability to detect, interpret, or respond to signals of sexual interest and cause challenges in intimate relationships. However, this hypothesis has not yet been verified. In the following sections, we will explore concepts which may be related to the ability or lack of it to adequately respond to social sexual cues.

## Broader sociocognitive context of schizophrenia

Deficits in social cognition in schizophrenia are well documented and strongly related to daily psychosocial functioning.^108^ Studies on people at high-risk states for developing psychosis suggest that mild sociocognitive deficit may be already present years before the start of full-blown psychosis.^109^^,^^110^ Impaired social cognition was also described in healthy siblings of patients,^111^ suggesting that this deficit might be a trait marker for schizophrenia. Social cognition in schizophrenia seems to be disrupted across several domains,^112^ especially in emotion processing, theory of mind, and empathy. Theory of mind, empathy, and emotion processing are interrelated cognitive functions that play crucial roles in interpersonal interactions.^113^^,^^114^

### Theory of mind

Theory of mind (ToM) is an ability to attribute mental states (such as beliefs or intentions) to oneself and other people.^115^ Development of mentalizing abilities starts already during childhood with a critical point around 4-5 years (when typically developing children start to understand the first order theory of mind)^116^ and continues through puberty to adolescence, when people gain the ability to use and comprehend white lies, irony, and sarcasm.^117^ These are skills that patients often struggle with, which is not only due to the so-called undermentalization (lack of ToM, similar to what we can see in autism) but also due to overmentalization (overattribution of mental states).^118^ These mentalization deficits often complicate communication, could lead to misunderstandings or odd social behavior, and make it hard to initiate and maintain relationships in the long term. Deficits in ToM are also related to alterations in corresponding brain areas (such as the temporoparietal junction or prefrontal cortex) and brain networks.^119^ According to Dodell-Feder et al.,^120^ ToM may be a key ingredient for the development and maintenance of healthy romantic relationships.

### Emotion processing

Emotion processing refers to the ability to effectively identify emotions in oneself and others. In schizophrenia, facial emotion attribution is the most researched area of emotional processing, however, deficits in emotional processing have been documented across all modalities (eg, voice^121^ or bodily movements^122^). Patients have significantly more difficulties to correctly identify emotions from facial expressions when compared to healthy controls.^123^ Some studies also reported overattribution of specific emotions (such as anger) to neutral faces in patients.^124^ Furthermore, fMRI studies show that during facial emotion processing, patients display hypoactivation in regions usually associated with emotion processing and increased activation in other areas (probably as a compensation mechanism).^125^

### Empathy

Empathy, defined as the ability to appreciate emotions of others with minimum self-other distinction,^126^ includes cognitive empathy (interpreting other people’s thoughts and feelings) and affective empathy (sharing other people’s emotions). Schizophrenia patients often lack both types.^127^ The ability to empathize supports higher relationship satisfaction^128^ and is one of the key skills necessary for higher social functioning.^127^ In recent years, the study of empathy has garnered increasing attention within the context of schizophrenia, particularly among individuals at high risk of developing the disorder.^129^^,^^130^ According to Kuis et al.,^129^ cognitive empathy is already impaired in the ultra-high-risk phase of psychosis. The findings of Montag et al.^130^ suggest that individuals at clinical high risk of psychosis show less emotional empathy than controls or individuals with schizophrenia, while individuals with schizophrenia show impaired cognitive empathy. Horton, Smith, and Haas^131^ conducted research clarifying early signs of social deficits in high-risk children and adolescents (ie, those with a family history of schizophrenia), revealing pronounced impairments in social skills, assertion, and empathy scores. These findings propose that deficits in this area may represent developmental precursors to the social amotivation domain of negative symptoms commonly associated with schizophrenia.

## Conclusion

The evidence suggests that both biological/hormonal and psychological/behavioral developmental trajectories in schizophrenia patients deviate from the normal course, potentially leading to sexual and romantic difficulties. This review has highlighted the close interconnection of biological, psychological, and social factors. Abnormalities in the HPG and HPA axes have been observed in schizophrenia patients, potentially affecting sociosexual competencies and resulting in behavioral difficulties. Deficits in theory of mind, emotion processing, and empathy may contribute to difficulties in forming and maintaining intimate relationships, further complicating the social challenges faced by individuals with schizophrenia. Early life traumas, such as emotional neglect and sexual abuse, may lead to social and sexual difficulties and insecure attachments, exacerbating the disorder.

The relationship between schizophrenia and sexual/romantic functioning appears to be bidirectional. Sexual and romantic difficulties may serve as early indicators of schizophrenia risk, while the disorder itself can exacerbate sexual and romantic functioning. The complex relationship underscores the importance of considering sexual and romantic functioning in the assessment, treatment, and support of individuals with schizophrenia. Future research should focus on further elucidating the mechanisms underlying sexual and romantic difficulties, especially in determining the emotional and motivational salience of sexual stimuli and capacity to engage in and maintain communication of sexual interest. Such efforts could potentially help to design sexological remediation strategies and improve overall quality of life, treatment adherence, and long-term outcomes for individuals living with this challenging disorder.
